# A 65–115 GHz VNA Extension Frontend Based on a High-Directivity Misaligned Waveguide Branch Coupler

**DOI:** 10.3390/mi17101159

**Published:** 2026-09-30

**Authors:** Yuchi Zhang, Bingli Dai, Zhongqian Niu, Ningbo Chen, Yinian Feng, Yonghong Zhang, Bo Zhang

**Affiliations:** 1 School of Electronic Science and Engineering, University of Electronic Science and Technology of China, Chengdu 611731, China; zyc1744833548@163.com (Y.Z.); daibingli@yeah.net (B.D.); fengyinian@uestc.edu.cn (Y.F.); zhangyhh@uestc.edu.cn (Y.Z.); 2 Shenzhen Institute for Advanced Study, University of Electronic Science and Technology of China, Shenzhen 518038, China; chenningbo@fesight.cn (N.C.); zhangbouestc@yeah.net (B.Z.)

**Keywords:** directivity, frequency extension, loose coupling, misaligned waveguide, vector network analyzer (VNA), waveguide branch coupler

## Abstract

The vector network analyzer (VNA) is the core instrument for S-parameter measurements in microwave and millimeter-wave bands. Frequency extension modules are indispensable for high-frequency testing, in which the directivity of the directional coupler directly determines the reflection measurement accuracy. To address the limited directivity and fabrication difficulty of conventional branch couplers in loose-coupling (10-dB) designs, this paper proposes a novel 12 dB waveguide branch coupler based on a misaligned structure and applies it to a 65–115 GHz VNA extension frontend. By introducing an offset in the branch waveguide, the coupler excites the TE_201_ mode and utilizes the resulting transmission zero to improve bandwidth and amplitude flatness. Meanwhile, the branch-height compensation mechanism introduced by the misaligned structure effectively alleviates the fabrication difficulty in loose-coupling designs and enhances directivity. Based on this coupler, a miniaturized extension frontend of only 60 × 60 × 30 mm^3^ is designed and implemented. The indirectly estimated directivity of the six-branch coupler is better than 16 dB over the 65–115 GHz band, and the module exhibits a dynamic range exceeding 100 dB at a 20 Hz IF bandwidth with an effective directivity better than 38 dB. Compared with mainstream commercial modules, the proposed design achieves comparable electrical performance while offering smaller size and lower fabrication complexity, providing an effective technical pathway for ultra-wideband, high-performance, low-cost, and integrable VNA frequency extension frontends in the millimeter-wave regime.

## 1. Introduction

The rapid advancement of millimeter-wave and terahertz technologies, driven by applications such as 6G communications and material characterization, has spurred an ever-growing demand for high-frequency device testing [1,2,3,4,5]. Concurrently, the increasing integration density and operating frequencies of monolithic microwave integrated circuits (MMICs) necessitate comprehensive S-parameter evaluation throughout both the research and production phases [6,7]. These trends pose significant challenges for test instrumentation in terms of frequency coverage, dynamic range, and, notably, physical footprint [8,9].

Among various high-frequency test instruments, the vector network analyzer (VNA) stands as the core tool for S-parameter measurements [10]. However, the coaxial operating frequency of commercial VNA mainframes is typically limited to 67 GHz, which is insufficient to fully cover the testing requirements in millimeter-wave and terahertz bands [11,12]. Developing VNA mainframes with higher native frequencies faces significant hurdles in terms of both technical complexity and manufacturing cost. Frequency extension modules, which upconvert the VNA’s RF and local oscillator (LO) signals to the target band through external multiplication and mixing chains, offer a critical means to overcome this limitation [13]. In recent years, photonic-based extension schemes have attracted attention due to their ultra-wideband coverage potential; however, their system complexity and high cost render their practicality still unproven [14]. Electronic extension schemes remain the mainstream approach in practical engineering applications. Further enhancement of their measurement accuracy hinges on the performance improvement of the directional coupler—the core component of such modules. This paper focuses on addressing this issue.

In electronic extension frontends, the directional coupler plays a critical role in separating the forward incident wave from the reverse reflected wave along the signal transmission path [15]. The directivity of the coupler directly determines the measurement accuracy of the system’s reflection parameters (S_11_/S_12_) [16]. Insufficient directivity causes leakage signals from the coupler to superimpose on the true reflected signals, causing the measured values of small reflection coefficients to deviate significantly from their actual values. Therefore, enhancing coupler directivity is the fundamental approach to improving the reflection measurement accuracy of extension modules.

As an alternative to the branch coupler, the multi-hole coupler represents another common form of waveguide directional coupler. Through the interference effect among multiple coupling holes, multi-hole couplers can achieve high directivity (typically 25–30 dB), and their structural characteristics naturally favor loose-coupling (e.g., 10-dB) designs. However, this type of coupler employs the H-plane (broad-wall) split-block assembly, and its configuration makes it difficult to integrate within the same cavity as other millimeter-wave circuits (such as mixers and frequency multipliers) that are laid out in the E-plane orientation [17,18]. In practical extension modules, the multi-hole coupler often has to be housed in a separate cavity and connected to the surrounding circuitry through additional waveguide transitions, which not only increases the module volume but also introduces extra assembly errors and insertion loss.

The waveguide branch coupler, in contrast, employs an E-plane split-block assembly, with its coupling structure oriented along the narrow-wall direction of the waveguide. This assembly approach is fully compatible with the cavity layout of active devices such as mixers and frequency multipliers in the millimeter-wave band, facilitating module miniaturization and high integration density [19]. However, branch couplers are predominantly used in tight-coupling (3 dB) scenarios, with very limited reports on loose-coupling (10 dB) designs—yet loose coupling is precisely the typical operating state of couplers in VNA extension modules (the coupled port must provide moderate input power to the mixer, while the insertion loss of the main line should be minimized). Under loose-coupling conditions, branch couplers face multiple conflicts among bandwidth, directivity, and manufacturability. Although Reed’s classical design theoretically established the foundation for branch couplers, the branch waveguide height decreases sharply in loose-coupling designs—often less than 0.15 mm in W-band 10 dB designs—resulting in not only fabrication difficulties but also limited bandwidth [20,21,22,23]. Rashid et al. extended the bandwidth by introducing corrugated structures, but at the cost of significant degradation in return loss and directivity [24,25]. The transmission-zero (TZ) technique previously proposed by our group successfully achieved ultra-wideband loose coupling in the terahertz band; however, its directivity was only on the order of 10 dB, which is insufficient for high-precision reflection measurements [26,27]. Therefore, how to simultaneously accommodate bandwidth, directivity, and manufacturability in loose-coupling branch couplers remains a critical issue to be addressed.

Our group recently proposed a novel branch waveguide coupler based on a misaligned waveguide structure and validated its effectiveness in improving amplitude imbalance in a V-band 3 dB coupler [28]. Building upon this misaligned structure, this paper extends it to a 10 dB coupling level and applies it to a 65–115 GHz VNA extension frontend. The proposed couplers and module are fabricated using high-precision computer numerical control (CNC) micromilling, which is a well-established micromachining technique for millimeter-wave waveguide devices. The specific contributions are as follows:

(1) Device-level: By introducing a central offset in the branch waveguide, the coupler excites the TE_201_ mode and utilizes the resulting transmission zero to broaden bandwidth while maintaining good directivity. Meanwhile, the branch-height compensation mechanism introduced by the misaligned structure effectively increases the minimum branch dimension in loose-coupling designs, significantly reducing fabrication difficulty.
(2) System-level: The designed 12 dB coupler serves as the core directional separation component and is integrated with active circuits—including frequency multipliers, amplifiers, and mixers—within a single E-plane split-block cavity, realizing a miniaturized extension frontend with a volume of only 60 × 60 × 30 mm^3^.
(3) Verification-level: Comprehensive experimental tests were conducted on both the coupler and the extension module. The coupler achieves a directivity better than 16 dB over the 65–115 GHz band, while the module exhibits a dynamic range exceeding 100 dB at a 20 Hz IF bandwidth and an effective directivity better than 38 dB. Compared with mainstream commercial modules, the proposed design achieves comparable electrical performance while offering smaller size and lower fabrication complexity. Moreover, its compact physical footprint facilitates integration with commercial probe stations, making it suitable for on-wafer S-parameter testing of millimeter-wave chips.

## 2. Design of the 12 dB Misaligned Waveguide Branch Coupler

**Figure 1:**
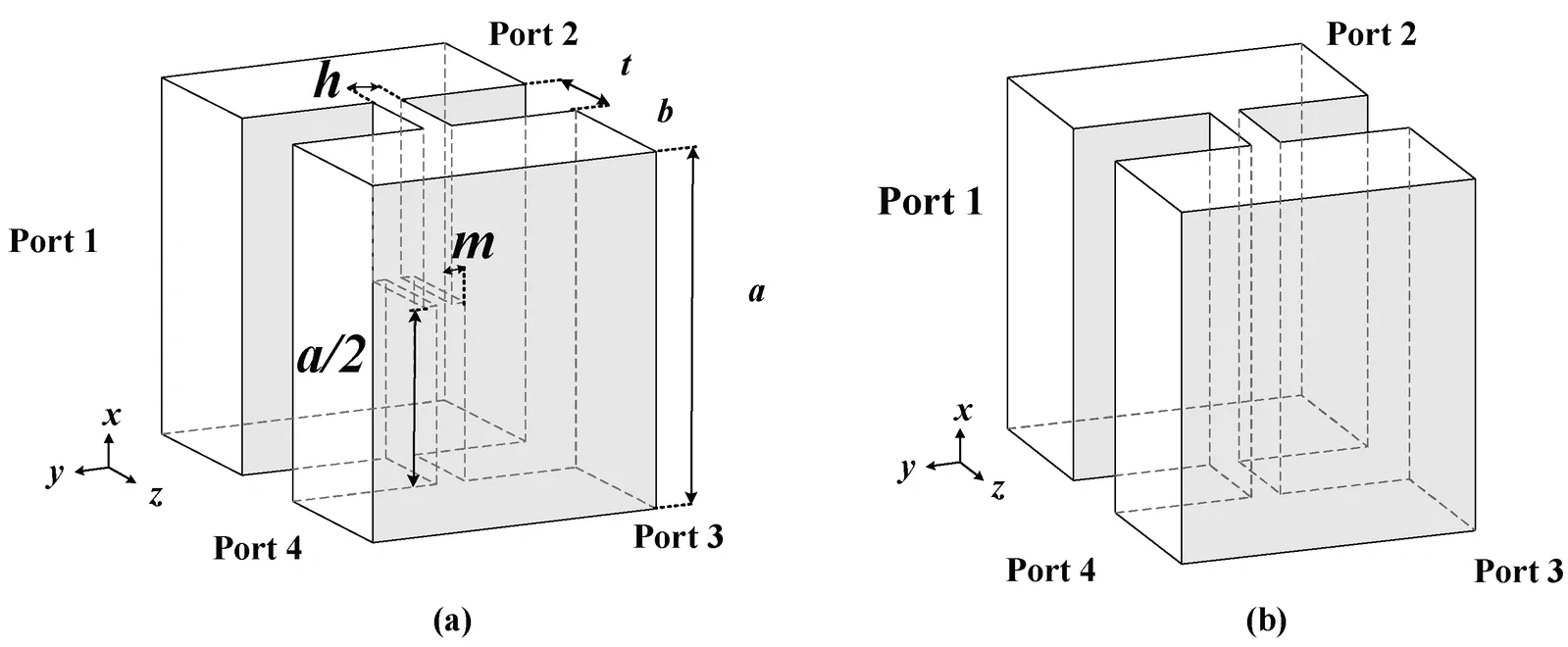
Schematic of the single-branch coupling structures: (**a**) misaligned waveguide-based branch coupler with key dimension parameters *a*, *b*, *h*, *m* and *t* labeled; (**b**) conventional Reed-type branch coupler.

Figure 1 illustrates a comparison between the single-branch coupling structure based on a misaligned waveguide and the conventional Reed-type branch coupler [20]. In the misaligned structure, a central offset *m* is introduced in the branch waveguide. The branch waveguide shares the same width *a* as the main waveguide, with its height denoted as *h* and its length as *t*. This offset breaks the structural symmetry, effectively exciting the TE_201_ mode. The transmission zero (TZ) generated by this mode enables the optimization of amplitude imbalance in loose-coupling designs [28].

**Figure 2:**
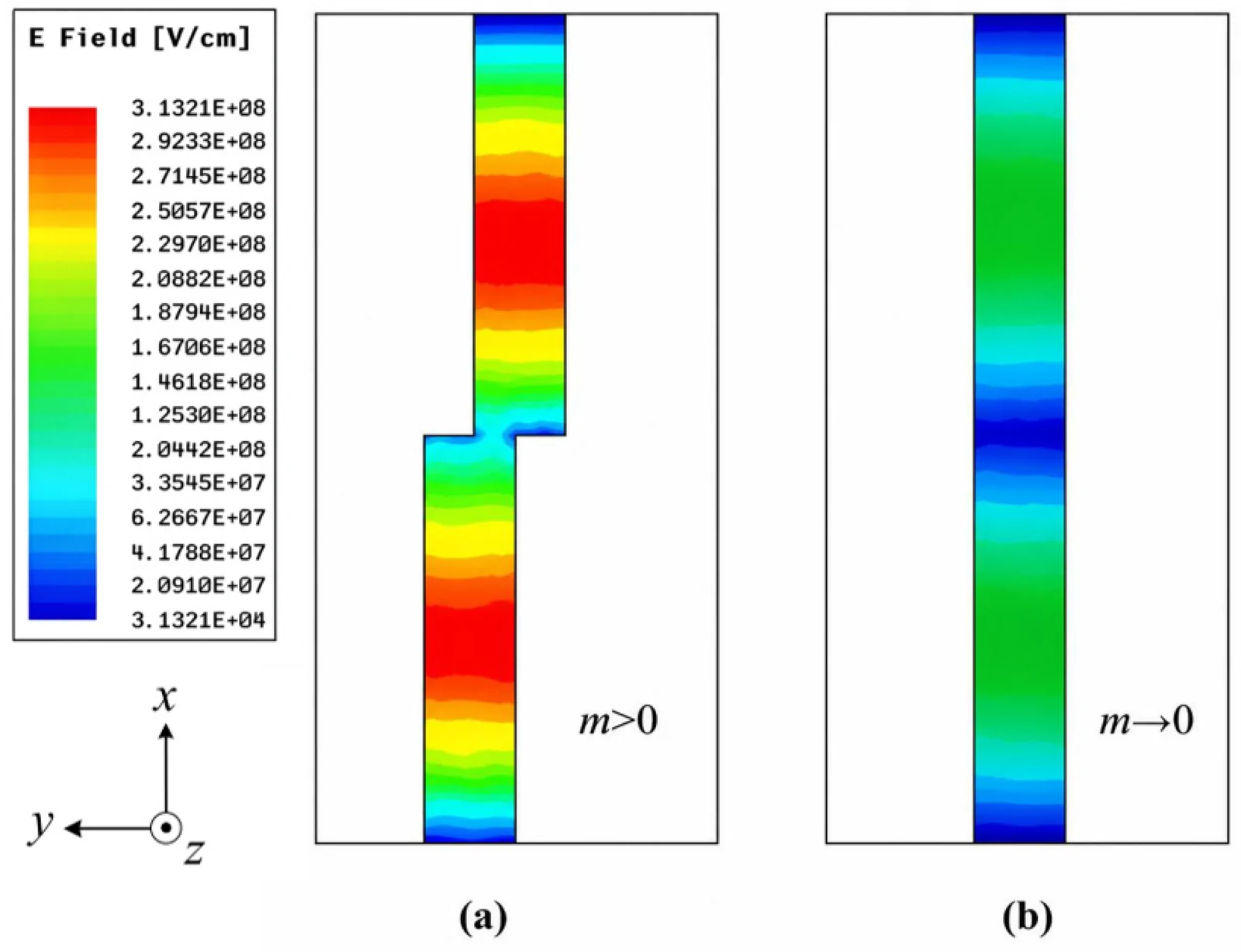
Electric field distribution of the TE_201_ mode in the proposed single-branch misaligned structure: (**a**) with a central offset *m* > 0, where the offset breaks the symmetry and excites the TE_201_ mode; (**b**) without offset (*m* → 0), where the mode is not excited.

To further investigate the operating mechanism of the proposed branch structure, an electric field analysis was conducted with respect to the offset *m*. The results indicate that even a small offset is sufficient to excite the TE_201_ mode (as shown in Figure 2), producing a TZ that measurably improves the coupling flatness. In the subsequent analysis, the TZ is utilized to improve the amplitude imbalance of the coupler.

It should be noted that the introduced offset inevitably excites other modes in addition to the TE_201_ mode, such as the TE_301_, TE_102_, and TE_202_ modes. However, the key advantage of the proposed misaligned structure lies in the fact that the resonant frequencies of these modes are all located outside the operating bandwidth of the coupler. This distinguishes the proposed design from the TE_301_-mode-cavity-based configuration analyzed in our previous work [28], in which unavoidable fabrication-induced asymmetry could potentially bring the TE_301_ resonance into the operating band. A detailed modal analysis is provided in [28].

**Figure 3:**
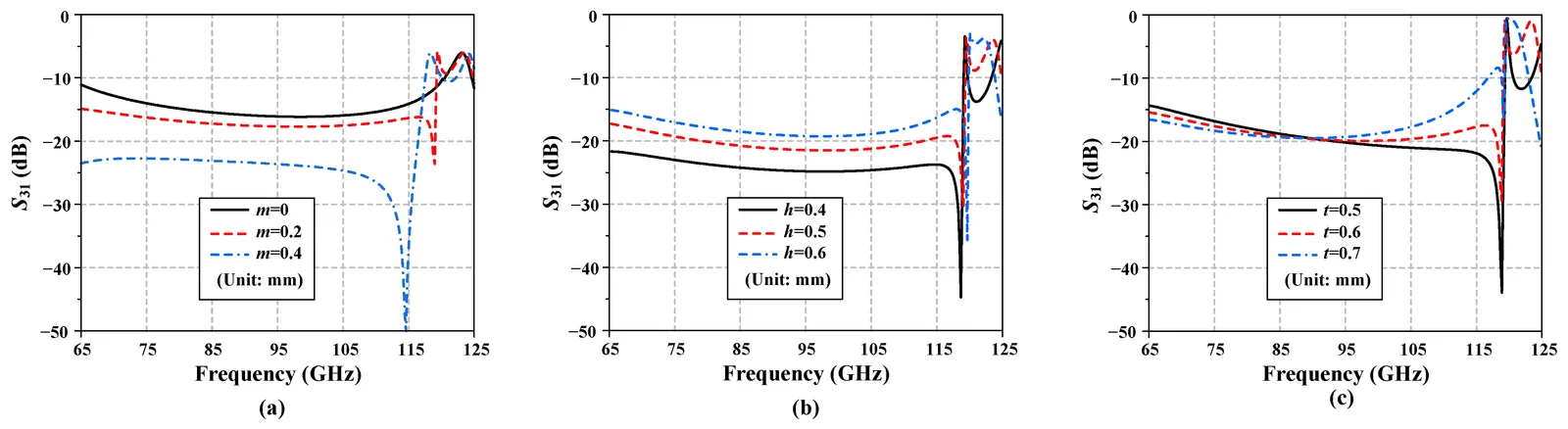
Effects of key dimensions on the *S*_31_ in the proposed single-branch misaligned structure: (**a**) offset *m*; (**b**) branch height *h*; (**c**) branch length *t*.

To establish the design basis, a parametric scanning analysis was conducted on the effects of key dimensions on the coupling coefficient. Figure 3a shows the influence of the offset *m* on the coupling coefficient. As *m* increases from 0 to 0.4 mm, the TE_201_ mode becomes increasingly pronounced, the coupling curve tends to flatten, and the coupling coefficient gradually decreases. Figure 3b presents the effect of the branch height *h* on the coupling coefficient. With increasing *h*, the coupling coefficient gradually increases, while the resonant mode remains essentially unchanged. Consequently, when the offset *m* increases, the coupling degree can be compensated by moderately increasing *h*, laying the foundation for alleviating fabrication difficulty in loose-coupling designs. Figure 3c illustrates the effect of the branch length *t* on the coupling response. Variations in *t* shift the center frequency, consistent with its role as a quarter-wavelength resonator at the design frequency. The above parametric characteristics provide the basis for the design methodology presented in the following subsection.

The coupling degree is generally determined by the characteristic impedance of the branch waveguide. The characteristic impedance of the conventional branch waveguide is given by

(1) $$Z_{0}=\frac{h_{0}}{a}\cdot \frac{Z_{\mathrm{TEM}}}{\sqrt{1-{\left(\lambda_{0}/2a\right)}^{2}}}$$

where *Z*_TEM_ = 120*π* Ω, *a* is the broad-wall dimension of the main waveguide, *h*_0_ is the initial branch height determined by Reed’s theory [20], and *λ*_0_ is the free-space wavelength at the center frequency.

For the misaligned branch waveguide, the structure can be equivalently treated as a rectangular waveguide with a broad-wall dimension of *a* + *m*, which has been validated in our previous work [28]. Its characteristic impedance is expressed as

(2) $${Z_{0}}^{\prime }=\frac{h}{a+m}\cdot \frac{Z_{\mathrm{TEM}}}{\sqrt{1-{\left[\lambda_{0}/(2(a+m))\right]}^{2}}}$$

where *h* is the height of the branch waveguide, and *m* is the central offset. By setting *Z*_0_′ = *Z*_0_, equating the characteristic impedance of the misaligned branch to that of the conventional branch, the compensation relationship between *h* and *m* can be derived as

(3) $$h=\frac{\sqrt{{\left(a+m\right)}^{2}-{\left(\lambda_{0}/2\right)}^{2}}}{\sqrt{a^{2}-{\left(\lambda_{0}/2\right)}^{2}}}\cdot h_{0}$$

Equation (3) provides the compensated branch height for a given offset *m*. The branch length *t* and the spacing between adjacent branches *d* are initially set to one-quarter of the guided wavelength at the center frequency:

(4) $$t=\frac{\lambda_{0}}{4\sqrt{1-{\left[\lambda_{0}/(2(a+m))\right]}^{2}}},\;d=\frac{\lambda_{0}}{4\sqrt{1-{\left(\lambda_{0}/2a\right)}^{2}}}$$

**Figure 4:**
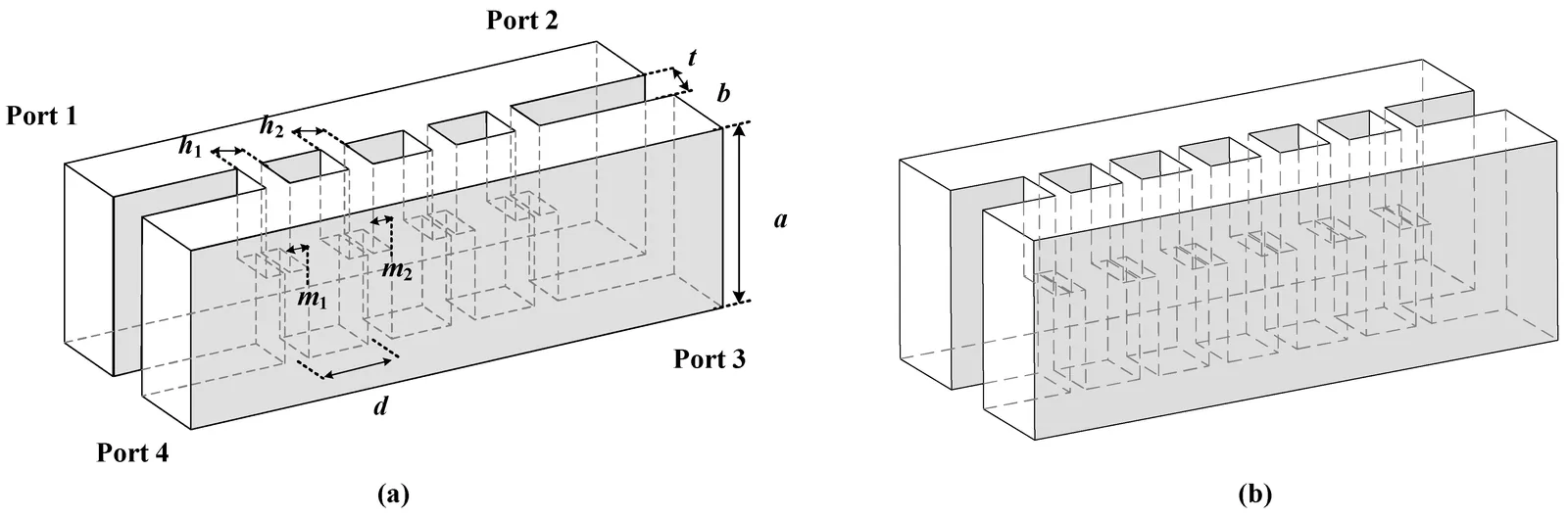
Schematic configurations of the two 12 dB misaligned branch couplers: (**a**) Four-branch structure; (**b**) six-branch structure.

Based on the design methodology described above, two 12 dB couplers were designed, with their configurations shown in Figure 4. The first design employs a four-branch structure, serving as a simplified verification vehicle to validate the effectiveness of the misaligned branch structure in loose-coupling scenarios. The second design utilizes a six-branch structure, which is the final configuration integrated into the VNA extension frontend. To more comprehensively evaluate the performance advantages of the proposed coupler, a conventional six-branch structure (*m* = 0) was also designed as a reference for comparison.

It is also worth noting that the number of branches plays a critical role in determining the coupler performance. As the number of branches increases, the frequency response approaches the ideal equal-ripple characteristic, which broadens the operating bandwidth and improves the coupling flatness. In addition, a larger number of branches leads to a more gradual impedance transformation along the coupling section, reducing the amplitude imbalance and improving the directivity, since the even- and odd-mode reflection coefficients become more closely matched. However, increasing the branch number also reduces the height and width of each individual branch, which significantly increases the fabrication difficulty. Therefore, the six-branch configuration is selected as a trade-off between electrical performance and manufacturability. A more detailed analysis of the relationship between branch number and coupler performance can be found in our previous work [28].

In the design process, the center frequency was set to 90 GHz, with the objective of achieving good coupling flatness and directivity over the 65–115 GHz frequency range.

The initial value was taken as *m* = *h*_0_, and *h* was then corrected using Equation (2). On this basis, electromagnetic (EM) simulations and the following parameter optimizations were further carried out.

(1) Optimize *t* and *d* to determine the center frequency of the coupler;

(2) Optimize *h* to achieve good amplitude flatness at the center frequency (target deviation within 1 dB);

(3) Optimize *m* to adjust the position and bandwidth of the TZ, further improving coupling flatness;

(4) Repeat steps (1)–(3) until all performance metrics satisfy the design requirements.

Following the above optimization procedure, the final geometric dimensions of the two couplers are summarized in Table 1. The four-branch coupler adopts the simplest configuration, with *h*_1_ = *h*_2_ and *m*_1_ = *m*_2_, and is designed for the 75–110 GHz band to serve as a verification vehicle for W-band systems. The two six-branch configurations—the conventional and misaligned designs—were then optimized based on Reed’s design approach to achieve improved directivity. The optimized parameters are summarized in Table 1.

**Figure 5:**
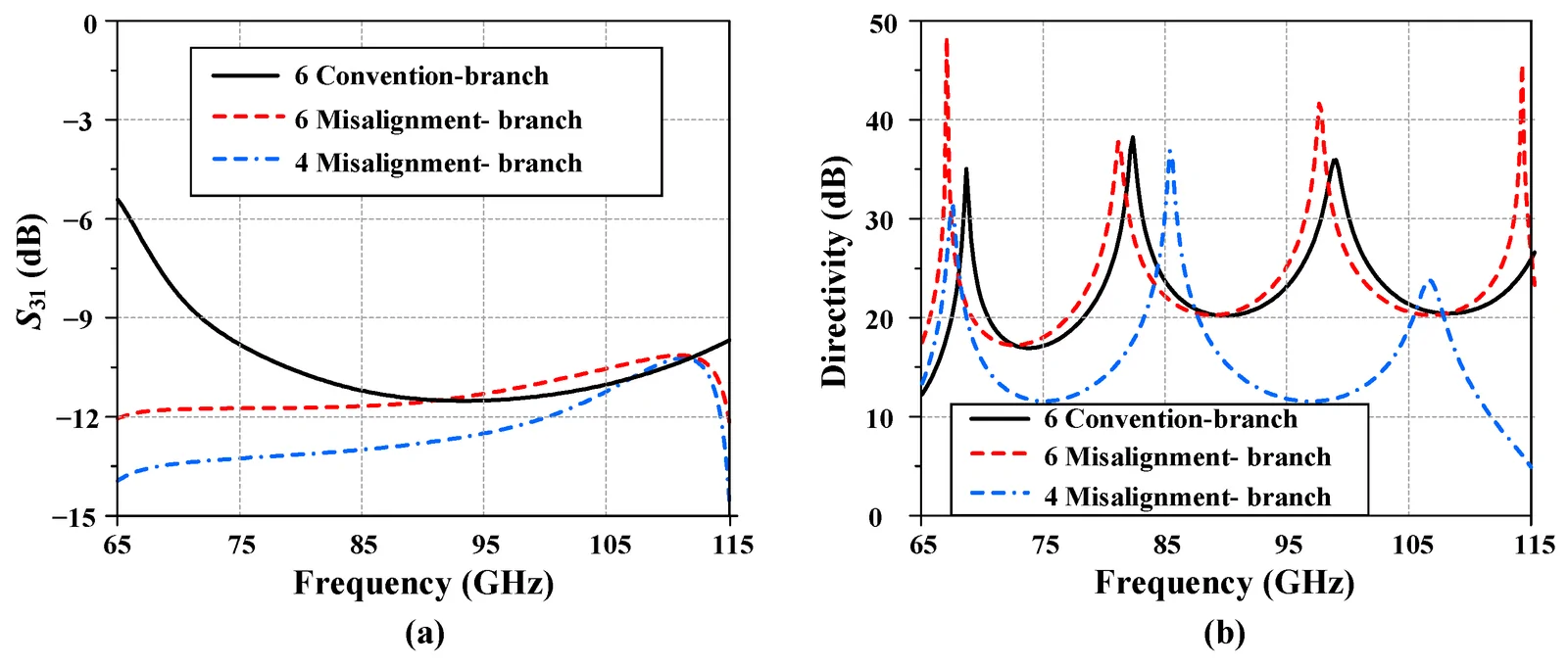
Simulated performance of the three couplers: (**a**) coupling responses; (**b**) directivity.

Figure 5a presents all simulated responses over 65–115 GHz for comparison, whereas the specified operating band of the four-branch coupler is 75–110 GHz. In the four-branch design, the coupling coefficient is approximately 13 dB with a coupling flatness better than 1.5 dB. In the six-branch misaligned design, the coupling flatness is improved to within 1 dB. For comparison, the conventional six-branch structure exhibits a sharp increase in coupling coefficient at low frequencies, and its branch height is only 0.11–0.14 mm, making it impractical for 65–115 GHz system applications in terms of both fabrication and performance. By contrast, the six-branch misaligned coupler achieves an *S*_31_ of approximately 11 dB with a flatness better than 1 dB, representing a significant improvement over its conventional counterpart.

Figure 5b presents the simulated directivity of the three couplers. Due to the limited number of branches, the directivity of the four-branch coupler begins to degrade above 110 GHz; nevertheless, it maintains a directivity of approximately 13 dB over the 75–110 GHz band, indicating its suitability as a core component for W-band VNA extension systems. The six-branch misaligned coupler achieves a directivity better than 17 dB across the ultra-wideband range of 65–115 GHz, with a directivity exceeding 20 dB over the 77–115 GHz sub-band. Meanwhile, its branch height exceeds 0.2 mm, which is approximately twice that of the conventional structure. These results demonstrate that the proposed structure not only enables wider bandwidth and reduced fabrication difficulty but also does so without sacrificing directivity.

The proposed couplers and the integrated extension module were fabricated using high-precision CNC micromilling, which is a widely adopted micromachining technique for millimeter-wave and terahertz waveguide devices. The machining accuracy was controlled within ±10 μm. More importantly, the proposed misaligned coupler features a larger branch height, which alleviates the challenge of CNC machining.

The waveguide cavity was split into upper and lower blocks along the E-plane. The two blocks were aligned using dowel pins and fastened with screws to ensure precise alignment between the branch waveguides and the main waveguide. This E-plane split-block assembly is fully compatible with the cavity layout of active millimeter-wave circuits such as mixers and frequency multipliers, enabling the coupler and active devices to be integrated into a single compact cavity. All subsequent cavities were fabricated using CNC milling. Among them, the four-branch coupler was individually fabricated and tested and then used to construct a discrete W-band VNA extension system for validation. The six-branch misaligned coupler, as the final design, was integrated into a compact extension frontend module. The measured performance of both couplers and the system-level test results will be presented in Section 4.

## 3. System Integration and Module Implementation of the Extension Frontend

### 3.1. System Architecture

**Figure 6:**
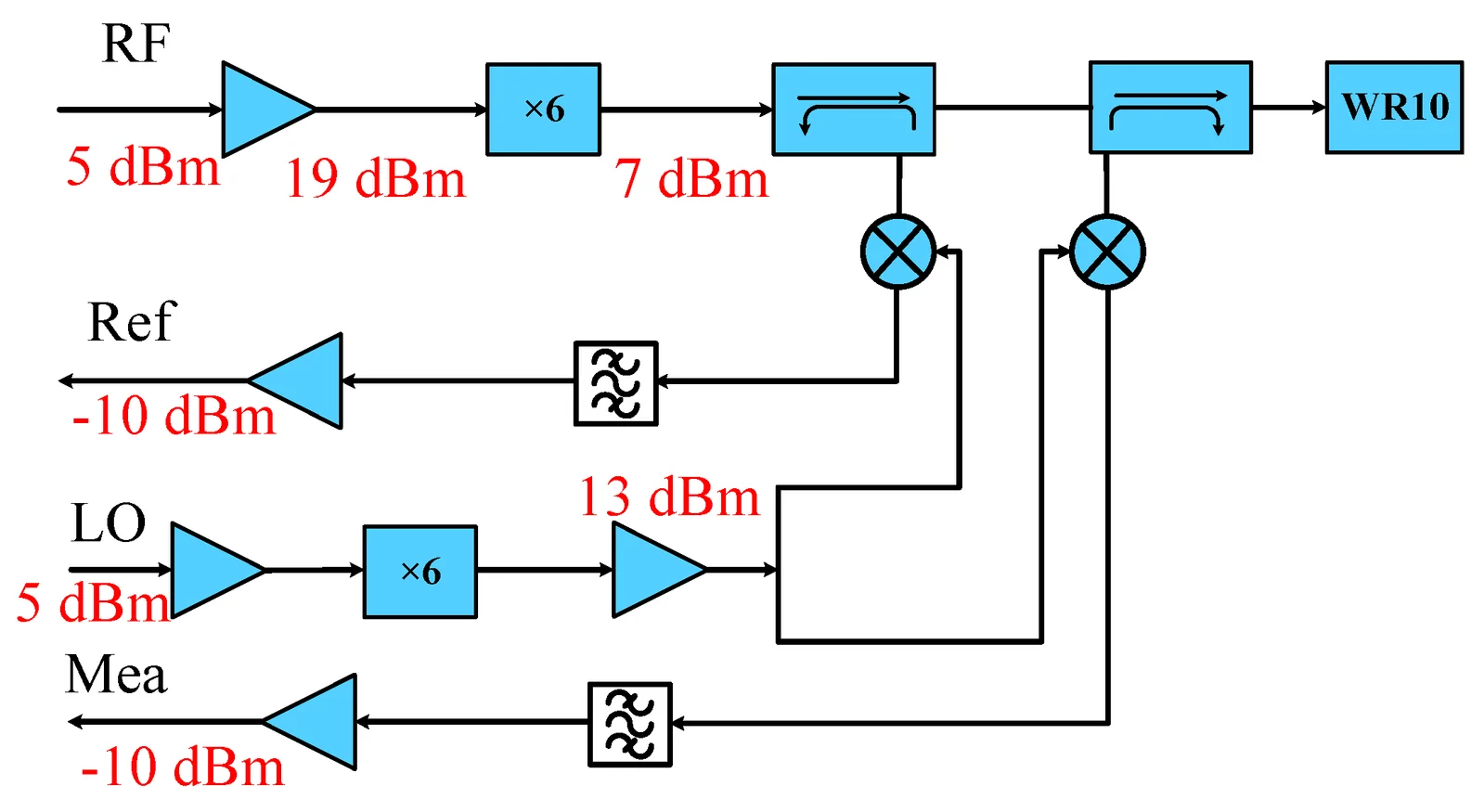
Block diagram of the extension module. RF: RF input; LO: local oscillator input; Ref: reference IF output; Mea: measurement IF output.

The extension module, whose block diagram is shown in Figure 6, adopts a frequency-multiplier-based transmit and superheterodyne receive architecture. The RF signal generated by the VNA mainframe (with a frequency range of approximately 12.5–18.33 GHz) is fed into the extension module through a coaxial interface. It is first amplified by a driver amplifier and then fed into a ×6 frequency multiplier, which multiplies the signal frequency to the W-band (75–110 GHz). Two identical six-branch misaligned couplers are integrated into the module. One coupler provides the reference signal (Ref), and the other detects the reflected signal (Mea). The two coupled signals are down-converted to intermediate frequencies (IF) by mixers and returned to the VNA mainframe through coaxial interfaces for further processing.

The LO signal generation chain is similar to that of the RF signal: the LO signal from the VNA mainframe (with a slight frequency offset from the RF signal) is amplified, multiplied by ×6, boosted to the required drive level by a power amplifier, and then divided by a power divider to the LO ports of the two mixers.

It is worth noting that loose coupling is adopted here to ensure that the mixers in the Ref and Mea channels operate well below their compression point, since conversion loss would otherwise vary with input power and introduce additional measurement errors. Based on this design consideration, two loose-coupling couplers were developed in this work, corresponding to two system configurations. The four-branch coupler, after being separately fabricated and tested, is used to construct a discrete extension system to verify the system-level directivity improvement. The six-branch coupler, as the final design, is directly integrated into the compact extension frontend module. These two configurations form a progressive relationship—the four-branch version serves to validate the feasibility of the mechanism, while the six-branch version realizes the final performance.

### 3.2. Preliminary System Verification with the Four-Branch Coupler

**Figure 7:**
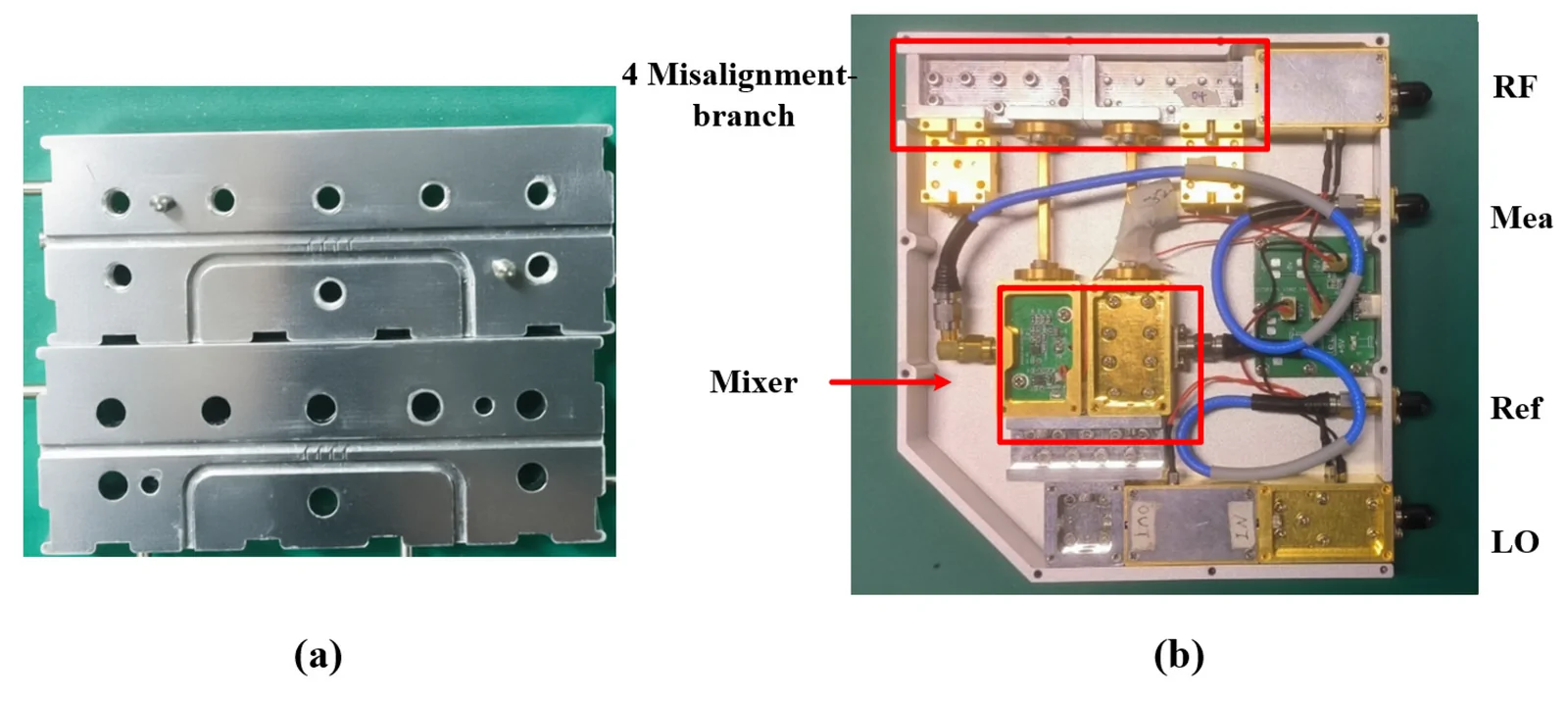
Photographs of the discrete extension system: (**a**) four-branch coupler; (**b**) overall connection configuration of the discrete system.

The four-branch coupler was separately fabricated and tested for S-parameter characterization. On this basis, a discrete extension system was constructed using the four-branch coupler, with its photograph shown in Figure 7. In this system, the functional modules—including frequency multipliers, couplers, mixers, and amplifiers—are cascaded in discrete form through waveguide and coaxial connectors. Although the discrete system is relatively bulky, its advantage lies in the interchangeability of each component, which facilitates the identification of performance bottlenecks and provides experimental guidance for the subsequent design of the integrated module. The measurement results will be presented in Section 4.

### 3.3. Integrated Six-Branch Module

**Figure 8:**
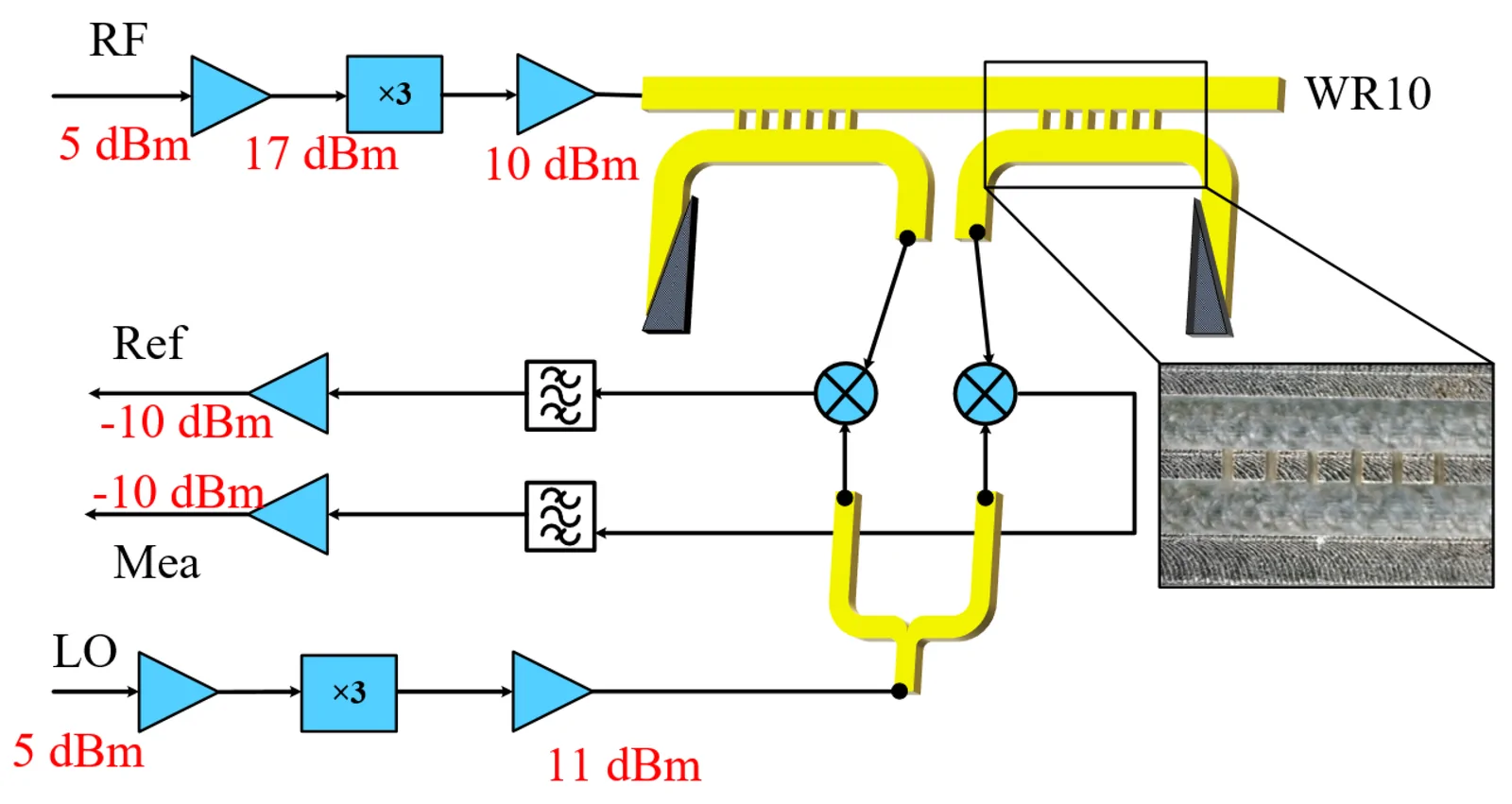
Internal block diagram of the integrated extension module and the local micrograph of the six-branch coupler.

The six-branch coupler, as the final design, was integrated into the compact extension frontend module. Figure 8 presents the internal block diagram of the integrated module, including the specific waveguide routing, cavity layout, and a local micrograph of the six-branch coupler. This diagram illustrates the physical layout within the module—including the spatial placement of each component, the routing of the waveguide transmission paths, and the cavity split-block strategy. Benefiting from this layout, the coupler shares the same E-plane split-block cavity with active devices such as frequency multipliers, mixers, and amplifiers, eliminating the need for additional waveguide transitions and thereby effectively reducing the module volume and assembly errors. In addition, the module adopts a unified Type-C interface for power supply to simplify the connector layout. The final module measures 60 × 60 × 30 mm^3^, which is significantly smaller than the discrete system. As described in Section 2, the integrated module was assembled using the same E-plane split-block approach, in which the upper and lower cavity blocks are aligned by dowel pins and fastened with screws.

## 4. Measurement Results and Discussion

### 4.1. Coupler Measurements

The four-branch misaligned coupler was fabricated using standard CNC micromilling with a machining accuracy of ±10 μm. S-parameter measurements were performed using a vector network analyzer with W-band extension modules, following a full two-port calibration.

Note that the four-branch coupler was designed for the 75–110 GHz band as a simplified verification vehicle for the misaligned structure in loose-coupling scenarios. After fabrication, it was tested using a commercial W-band VNA extension module and subsequently employed in a 75–110 GHz discrete extension system as a laboratory instrument. Only after the concept was successfully validated over this band did we proceed to the 65–115 GHz design for the final integrated module. Therefore, the measurement of the four-branch coupler is intentionally limited to its design band of 75–110 GHz.

**Figure 9:**
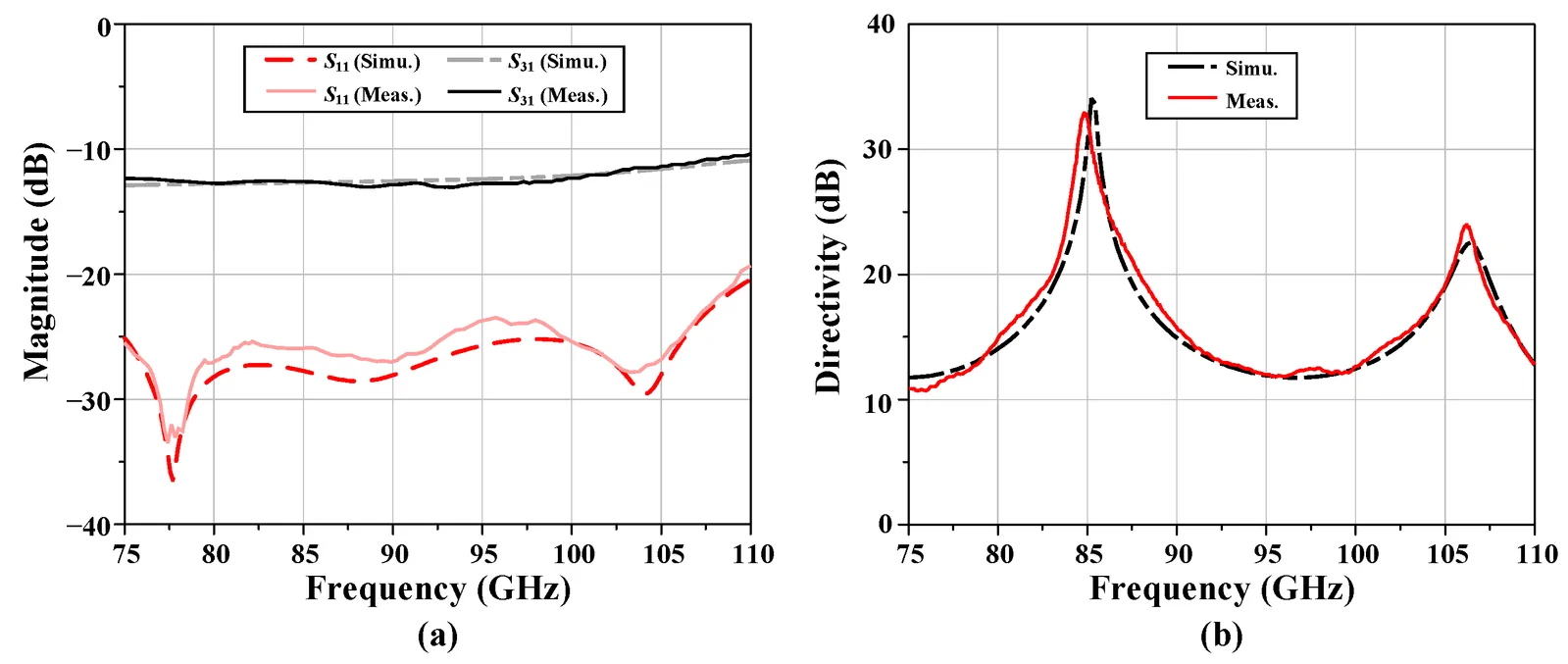
Simulated and measured results of the four-branch misaligned coupler: (**a**) S-parameters; (**b**) directivity.

Figure 9a presents the measured S-parameters of the four-branch coupler over the 75–110 GHz band, together with the simulated results for comparison. The measured coupling coefficient is approximately 13 dB with an in-band ripple of less than ±1.5 dB, showing good agreement with the simulated result. The reflection coefficient is better than −19 dB across the entire band.

Figure 9b shows the measured directivity. The four-branch coupler achieves a directivity better than 11 dB over the 75–110 GHz band, slightly lower than the simulated value of 13 dB. This minor discrepancy is primarily attributed to fabrication tolerances—particularly the influence of the ±10 μm machining accuracy on the offset *m* and branch height *h*, as discussed in the tolerance analysis of our previous work. Nevertheless, the measured directivity curve maintains good consistency with the simulation and surpasses the TE_301_-based cavity design (~10 dB), validating the effectiveness of the misaligned structure in loose-coupling and ultra-wideband applications.

**Figure 10:**
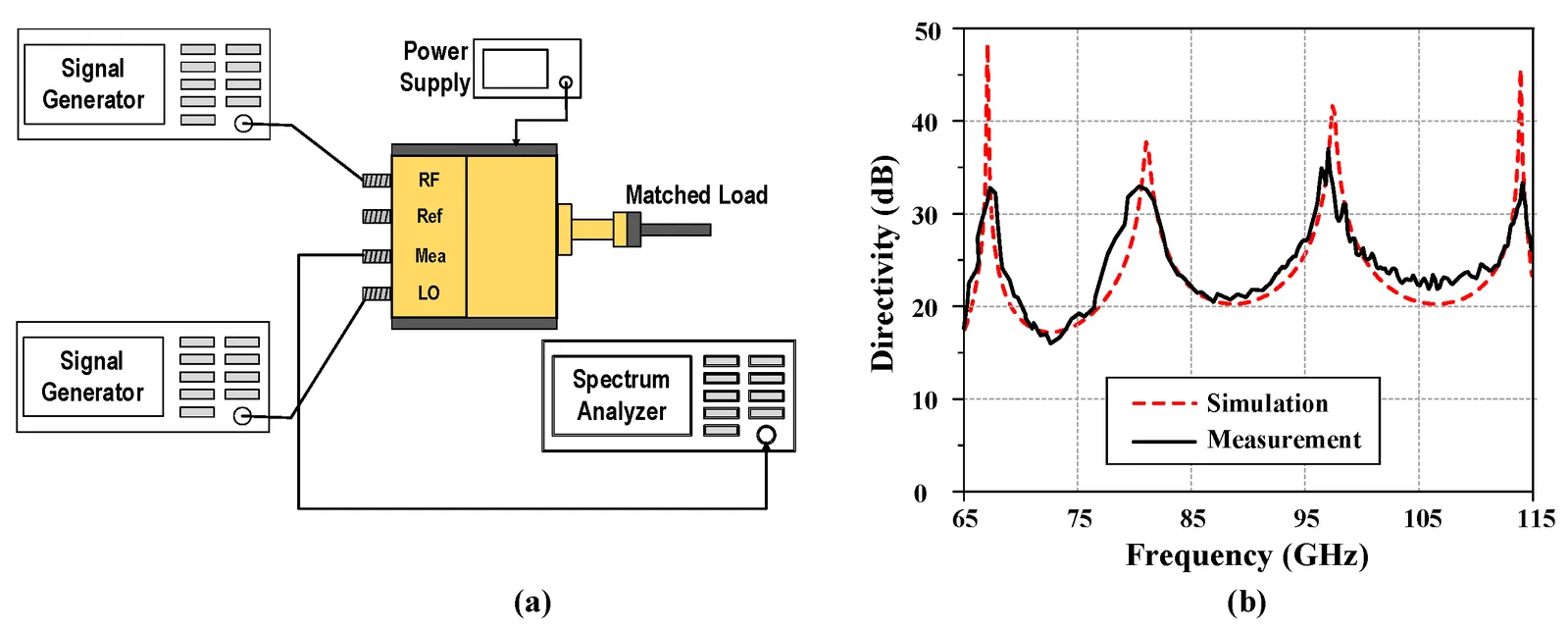
Indirect measurement of the six-branch coupler directivity: (**a**) test setup block diagram; (**b**) extracted directivity versus frequency.

The six-branch coupler was not fabricated and tested as a standalone device—its coupled ports are not readily accessible for direct S-parameter measurements via probes or coaxial interfaces. Alternatively, the directivity of the coupler can be extracted indirectly using the test method illustrated in Figure 10a.

The signal source provides separate RF and LO excitation signals. With the WR-10 port terminated by a matched load, the output powers at the reference IF port (Ref) and the measurement IF port (Mea) are recorded. Subtracting the Mea port power from the Ref port power yields a value approximately equal to the sum of the coupler directivity and insertion loss. By estimating the insertion loss as approximately 1 dB, the extracted directivity shown in Figure 10b is obtained. The indirectly extracted directivity of the six-branch coupler is better than 16 dB, which is in reasonable agreement with the simulated value of better than 17 dB. The observed fluctuations between the measured and simulated results are mainly attributed to fabrication errors and the performance mismatch between the two IF chain components.

On the other hand, the actual performance of the six-branch coupler can also be indirectly validated through the module’s effective directivity and dynamic range—both of which are directly influenced by the coupler directivity. These system-level results will be presented later.

### 4.2. System Testing of the Four-Branch Coupler-Based System

To validate the system-level directivity improvement enabled by the misaligned coupler, a discrete VNA extension system was constructed using the four-branch coupler. The VNA mainframe provides RF and LO signals, which are multiplied to the W-band by ×6 frequency multipliers. The RF signal passes through the four-branch coupler, where the forward and reflected signals are coupled out separately, down-converted to IF by mixers, and then returned to the VNA for processing.

Before each measurement session, a full two-port TRL calibration was performed using custom-designed through, reflect (short), and line standards, with the reference plane set at the waveguide flange interfaces of the DUT ports.

**Figure 11:**
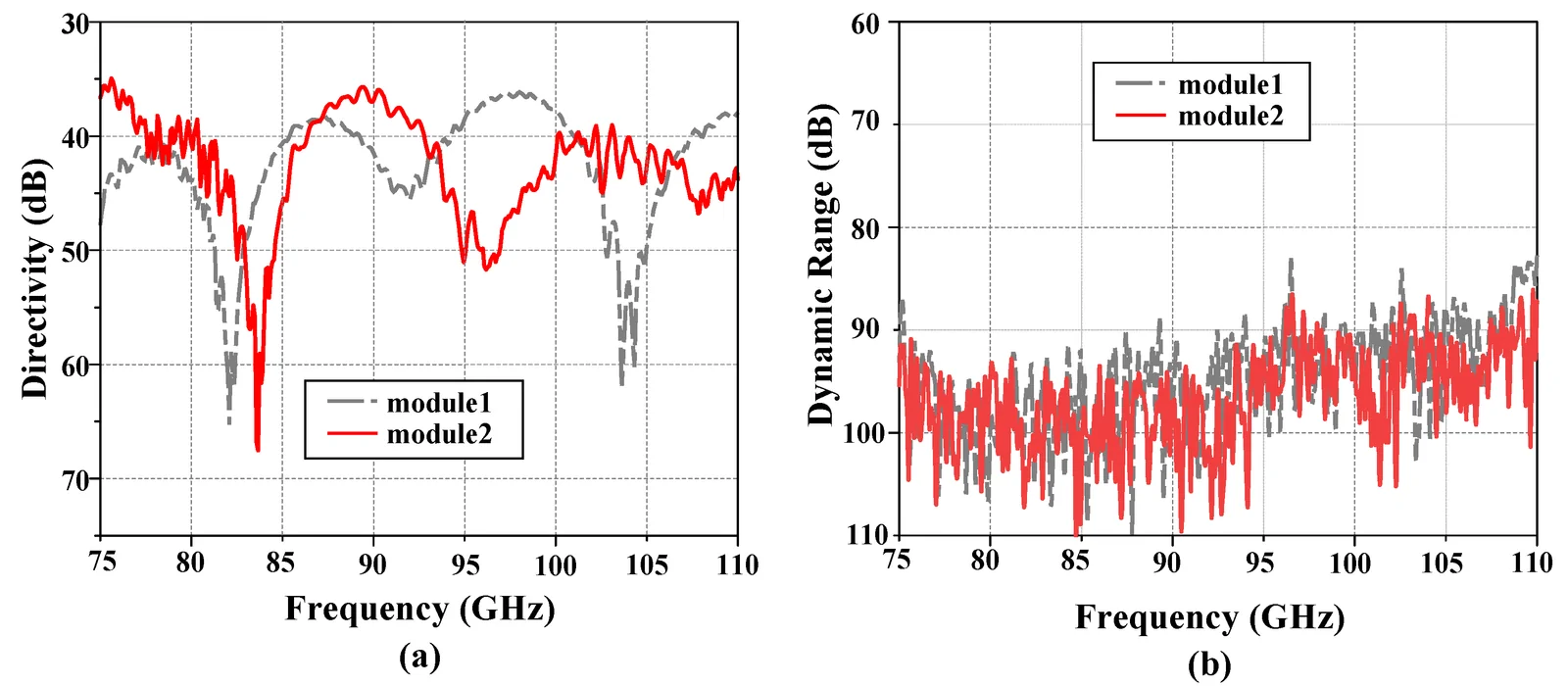
Measured results of the discrete four-branch extension system: (**a**) effective directivity; (**b**) dynamic range.

The system effective directivity was obtained by connecting matched loads to both ports after calibration and recording the *S*_11_ and *S*_22_ traces. Figure 11a presents the measured effective directivity of the discrete four-branch system. The system achieves an effective directivity better than 32 dB over the 75–110 GHz band. This value represents the calibrated residual effective directivity obtained after applying the VNA error-correction algorithm.

Although the four-branch coupler alone does not exhibit exceptional directivity, the well-designed system layout results in an effective directivity of 32 dB for the extension module, which is comparable to that of conventional VNA extension modules (typically ~30 dB), thereby validating the feasibility of the overall approach.

The system dynamic range was obtained by connecting matched loads to both ports after calibration and recording the *S*_21_ and *S*_12_ traces with a 20 Hz IF bandwidth. Figure 11b presents the measured dynamic range of the discrete four-branch system over the 75–110 GHz band. The system achieves a dynamic range better than 80 dB across the entire band, which is comparable to that of commercial W-band extension modules (typically 80–100 dB).

The dynamic range is primarily determined by the RF output power, coupler insertion loss and coupling factor, mixer conversion loss, and the VNA noise floor. The measured dynamic range confirms that the four-branch coupler does not significantly degrade system sensitivity.

**Figure 12:**
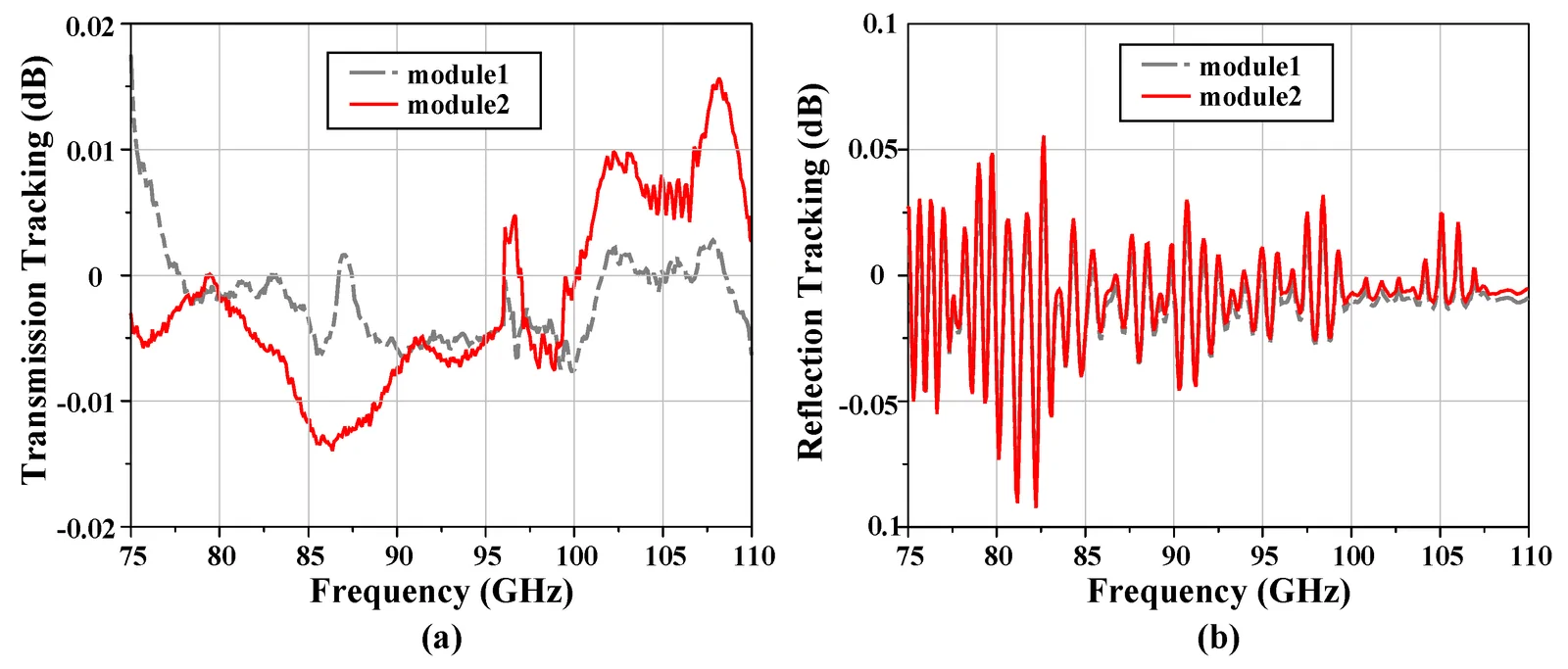
Tracking performance of the discrete four-branch extension system: (**a**) transmission tracking; (**b**) reflection tracking.

Transmission tracking and reflection tracking are key indicators for evaluating the amplitude and phase consistency between the reference and test channels of the system. After calibration, transmission tracking was evaluated by measuring a through connection (waveguide flange-to-flange connection) and recording the *S*_21_ and *S*_12_ traces. Figure 12a presents the transmission tracking results of the discrete four-branch system. The amplitude ripple is less than ±0.02 dB across the entire band, indicating good frequency-response flatness between the reference and test channels.

Reflection tracking was evaluated by connecting short standards to both ports after calibration and measuring *S*_11_ and *S*_22_. Figure 12b presents the reflection tracking results. The amplitude ripple is less than ±0.09 dB across the entire band, demonstrating good consistency between the incident and reflected signal paths.

### 4.3. Integrated Six-Branch Module Performance

**Figure 13:**
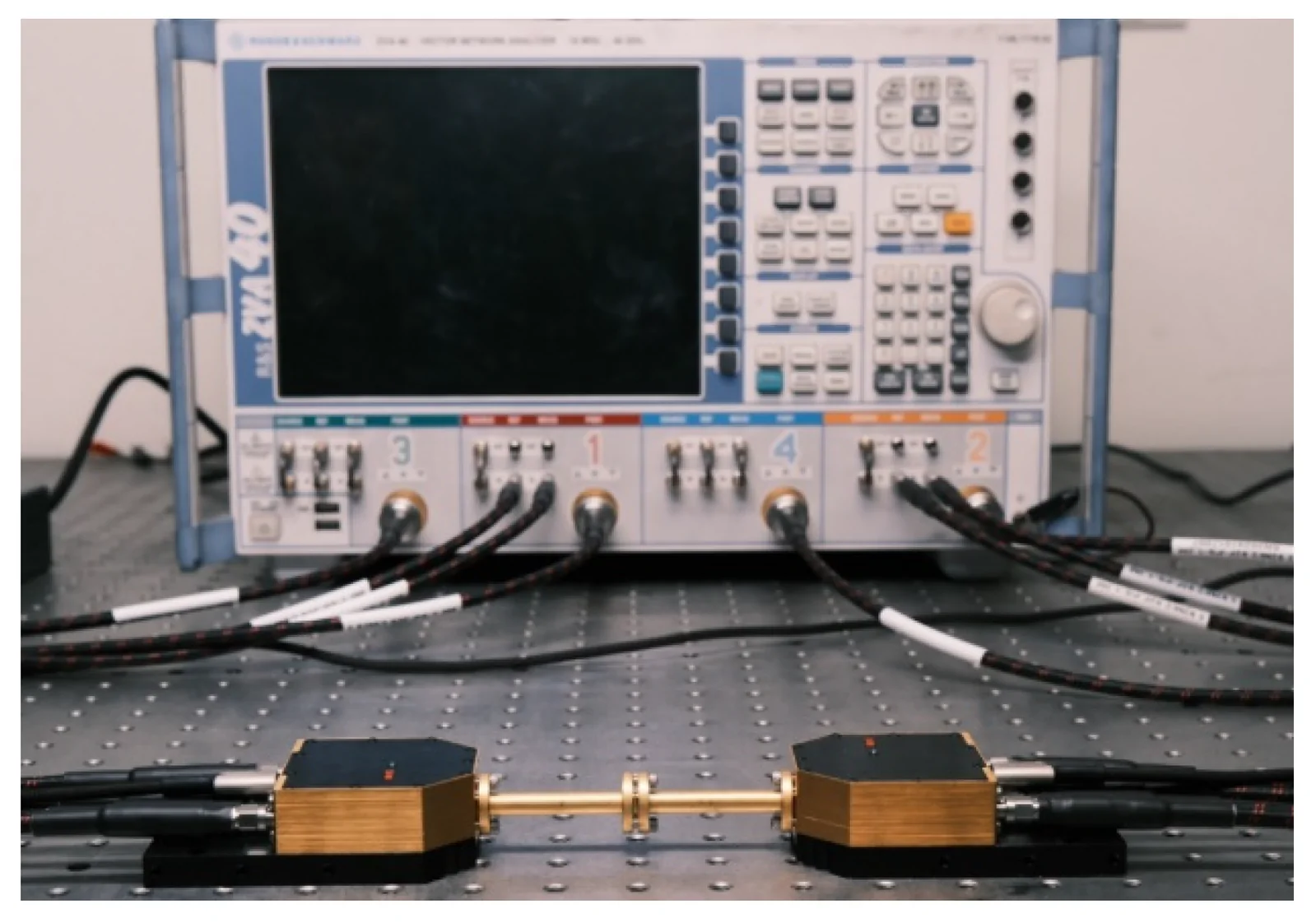
Actual test scenario of the integrated six-branch extension module connected to the VNA mainframe.

The integrated six-branch module was assembled following the layout described in Section 3.3. Figure 13 shows the test scenario of the integrated six-branch extension module. The module integrates two six-branch misaligned couplers, frequency multipliers, amplifiers, and mixers within a single E-plane split-block cavity, with overall dimensions of 60 × 60 × 30 mm^3^. The measurement setup is identical to that used for the discrete system, with the VNA mainframe providing RF and LO signals to the integrated module.

Before each measurement session, a full two-port TRL calibration was performed using the same calibration standards, with the reference plane set at the waveguide flange interfaces of the DUT ports of the integrated module.

**Figure 14:**
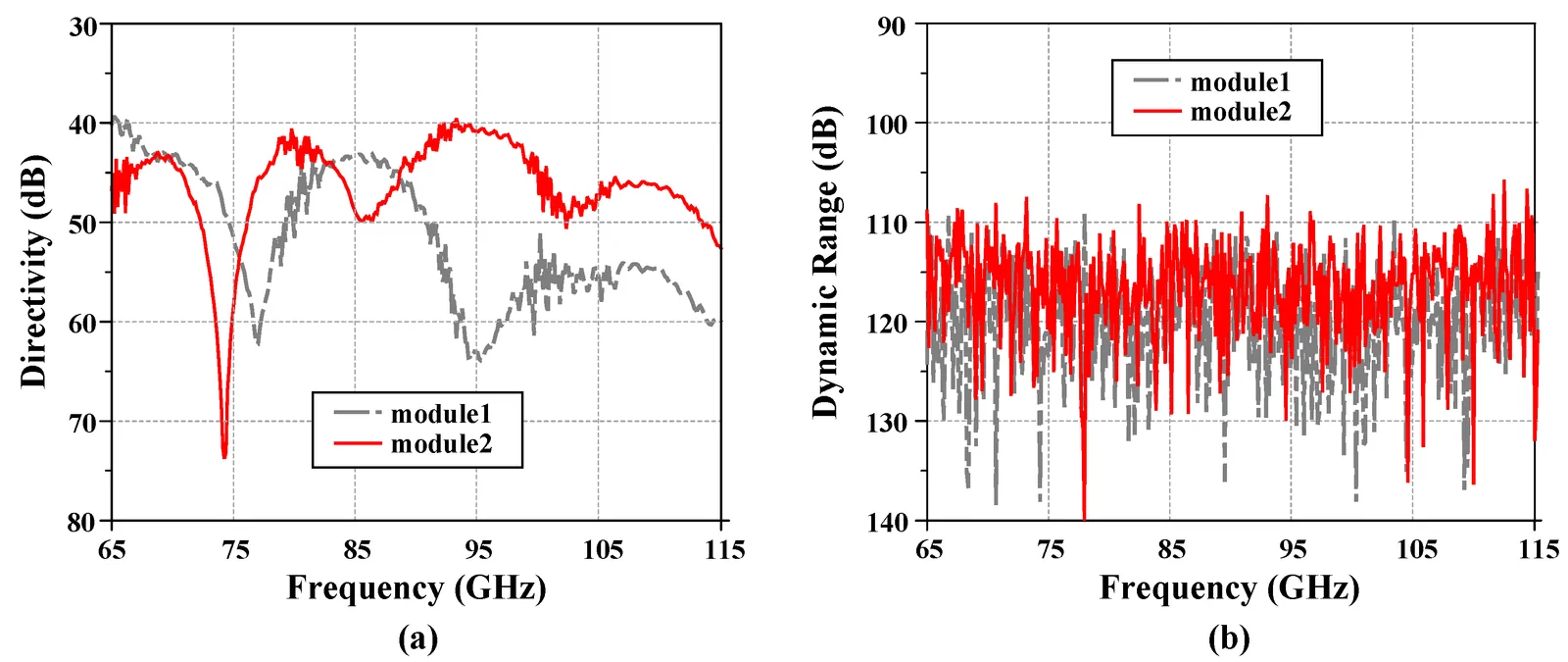
Measured results of the integrated six-branch module: (**a**) effective directivity; (**b**) dynamic range.

Figure 14a shows the measured effective directivity of the integrated six-branch module. The module achieves an effective directivity better than 38 dB over the 65–115 GHz band, which is approximately 6 dB higher than that of the discrete four-branch system.

The improvement in effective directivity over the discrete system can be attributed to several factors: the six-branch design inherently offers higher directivity than the four-branch design; the E-plane integrated assembly eliminates the waveguide interconnections that caused impedance mismatches in the discrete system, reducing signal leakage paths that degrade directivity; and the integrated terminations of the unused coupler ports are better matched than the discrete terminations used in the preliminary system.

Figure 14b presents the measured dynamic range of the integrated six-branch module with a 20 Hz IF bandwidth. The module achieves a dynamic range better than 100 dB across the entire band, which represents an improvement of approximately 20 dB over the discrete four-branch system (better than 80 dB). This improvement is primarily attributed to the following factors: the six-branch coupler exhibits lower insertion loss and a flatter coupling response compared with the four-branch design, and the E-plane integrated assembly eliminates waveguide transitions and interconnections, reducing signal reflections and insertion loss. Although this dynamic range still falls short of that of commercial W-band extension modules from leading manufacturers such as VDI (≥110 dB) and R&S (≥110 dB), it already demonstrates preliminary practical viability.

**Figure 15:**
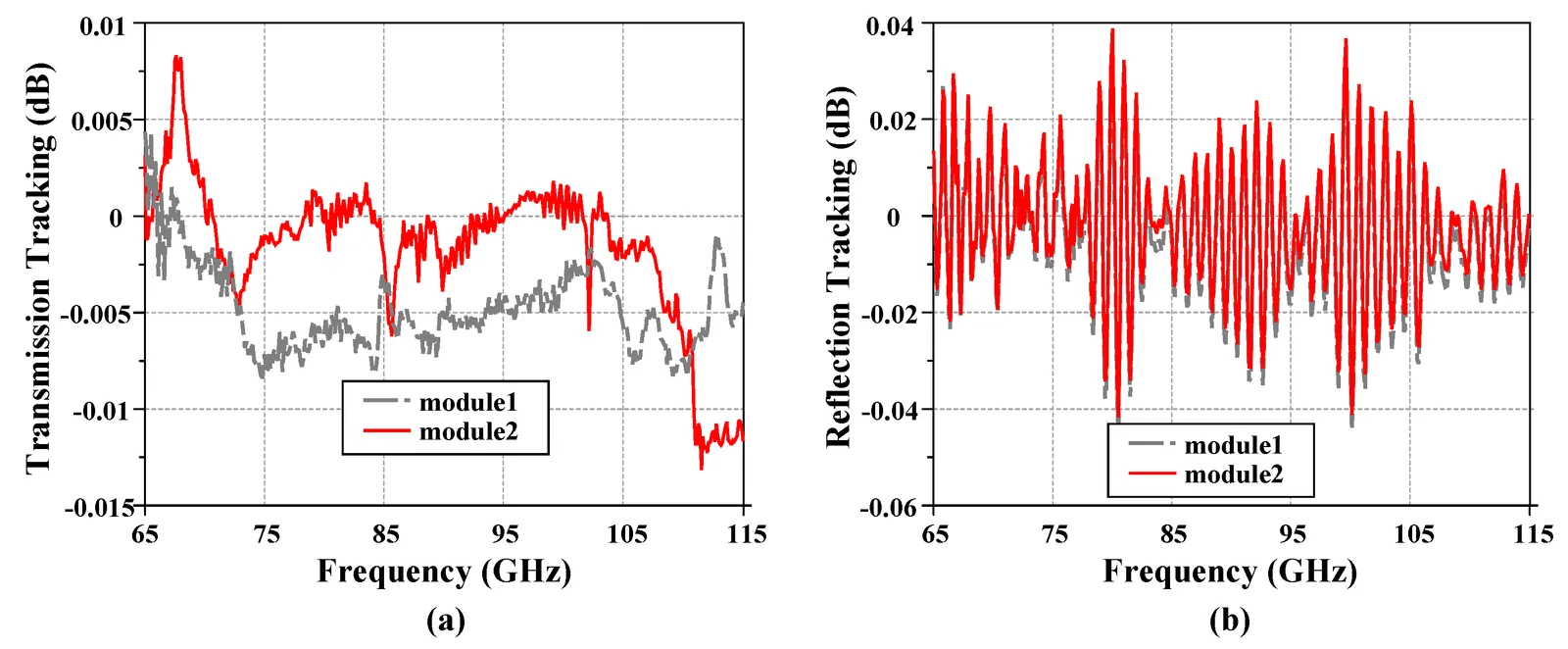
Tracking performance of the integrated six-branch module: (**a**) transmission tracking; (**b**) reflection tracking.

Figure 15a presents the transmission tracking results of the integrated six-branch module. The amplitude ripple is less than ±0.01 dB across the entire band, which is superior to that of the discrete system (±0.02 dB). This improvement is attributed to the elimination of interconnection mismatches in the integrated assembly.

Figure 15b shows the reflection tracking results. The amplitude ripple is less than ±0.05 dB across the entire band, also outperforming the discrete system (±0.09 dB). The improved reflection tracking indicates that the integrated assembly provides better impedance consistency between the incident and reflected signal paths.

## 5. Conclusions

This paper has presented a miniaturized 65–115 GHz VNA extension frontend based on a misaligned waveguide branch coupler, effectively addressing the inherent trade-offs among directivity, manufacturability, and system integration in loose-coupling branch couplers.

Two coupler variants were designed: a four-branch version for mechanism verification and a six-branch version for final module integration. The four-branch coupler achieved a directly measured directivity better than 11 dB, whereas the six-branch coupler exhibited an indirectly estimated directivity better than 16 dB—significantly outperforming conventional designs. The misaligned structure improves directivity by exciting the TE_201_ mode to generate a transmission zero, while the offset compensation mechanism increases the minimum branch height, thereby reducing fabrication difficulty.

The six-branch coupler was integrated into a 60 × 60 × 30 mm^3^ E-plane split-block module, which is substantially smaller than most commercial products. System-level measurements show that the module achieves a dynamic range better than 100 dB (20 Hz IF bandwidth), an effective directivity better than 38 dB over the 65–115 GHz band.

In summary, the misaligned waveguide structure effectively resolves the multi-objective trade-offs in loose-coupling branch couplers, and the E-plane integration strategy enables compact high-performance VNA extension modules. The fabrication approach based on high-precision CNC micromilling is well-suited for millimeter-wave and terahertz waveguide devices, offering a practical pathway for low-cost microsystem integration.

## Figures and Tables

**Table 1:** Optimized dimensions of the three 12 dB couplers (unit: mm).

Dimension	6 Convention-Branch	4 Misalignment-Branch	6 Misalignment-Branch
*a*	2.54	2.54	2.54
*b*	1.27	1.27	1.27
*h*_1_	0.11	0.36	0.22
*h*_2_	0.14	0.36	0.25
*m*_1_	0	0.28	0.19
*m*_2_	0	0.28	0.22
*t*	0.79	0.75	0.79
*d*	0.95	1.05	0.97

## Data Availability

The original contributions presented in this study are included in the article. Further inquiries can be directed to the corresponding author.
